# The Congress of American Physicians and Surgeons

**Published:** 1894-06

**Authors:** 


### Editorial.

The Congress of American Physicians and Surgeons was held at Washington City during the four days of May 29th, 30th, 31st, ending with June 1st, and presented many features of interest to the medical profession. The honor of having initiated the organization belongs to Dr. C. H. Mastin, of Mobile, Ala., and he was toasted as the father of the Congress at the banquet on the evening of the 30th of May, to which he responded in a speech eminently suited to the occasion.

This body is composed of the union of the several special associations once in three years, and these associations hold their separate meetings during the forenoon and join together in the Congress during the afternoon of each day. There are now fourteen different organizations of those devoted principally to the respective fields of work indicated by their names, as follows: American Ophthalmological Society, American Otological Society, American Neurological Society, American Gynecological Society, American Dermatological Association, American Laryngological Association, American Climatological Association, Association of American Physicians, American Association of Genito-urinary Surgeons, American Orthopedic Association, American Physiological Society, Association of American Anatomists, American Pediatric Society and American Surgical Association.

The meetings of the Congress with the constituent organizations are held in Washington City, as stated above, every three years, as provided by the constitution, but a proposition was made at the recent meeting to provide for holding meetings elsewhere, and has to lie over for consideration until the next meeting.

The separate societies and associations hold their meetings annually at such points as may be selected, and as the membership is largely from the Northern and Western States, there is but little expectation of having a meeting of either of these organizations in a

Southern State. It is to be hoped that more of our representative men may enter upon the advanced scientific plane of these special organizations and make their influence felt by the profession. This is another wide field inviting the coöperation of medical men, and meets the requirements of a large proportion of practitioners in these several departments. The obstacles to entrance in these special organizations by men of recognized status in their spheres of duty are readily overcome, and we have duly enrolled in the American Surgical Association Drs. C. H. and W. McD. Mastin, Briggs, McGuire, Tiffany, Miles, Yandell, Halsted, Michael and Smith, while other prominent Southern members of the profession belong to some of these societies above named.

The writer was complimented at the meeting in Buffalo by election to fellowship in the American Surgical Association, and read an elaborate paper upon "Mooted Points as to the Fractures of the Arm," etc., at the recent meeting in Washington. This paper will be published in an early issue of the *Medical News* of Philadelphia, and having the promise of reprints of it, any one desiring to have a copy can be supplied upon application.

The character of the work done in this branch of the Congress was most instructive, and the opening paper by Dr. John Ashhurst, Jr., of Philadelphia, upon "Empyema" covered the ground which has been explored in different parts of the world during the last decade. Some of the points presented met with criticism in the general discussion which ensued by the fellows of the Association, and when all the remarks are published with the transactions, the profession will have a very full report of the different phases of this important and serious disorder.

The writer contributed his mite, by drawing attention to the tendency of thoracic abscess to the anterior portion of the chest, and noted two cases in which he observed the spontaneous discharge of empyema near the sternum. J. MCF. G.

---

THERE is a chance for some one to discover a cure for the methylbenzonethoxyethyletetrahydrophyridinecarboxylate habit.—*Ex.*

